# Impacts of ribosomal RNA sequence variation on gene expression and phenotype

**DOI:** 10.1098/rstb.2023.0379

**Published:** 2025-03-06

**Authors:** Griffin A. Welfer, Ryan A. Brady, S. Kundhavai Natchiar, Zoe L. Watson, Emily J. Rundlet, Jose L. Alejo, Anand P. Singh, Nitish K. Mishra, Roger B. Altman, Scott C. Blanchard

**Affiliations:** ^1^ Department of Structural Biology, St. Jude Children’s Research Hospital, Memphis, TN 38105, USA; ^2^ Department of Molecular Biosciences, The University of Texas at Austin, Austin, TX 78712, USA; ^3^ Department of Chemical Biology & Therapeutics, St. Jude Children’s Research Hospital, Memphis, TN 38105, USA

**Keywords:** ribosome, translation, transcription, rDNA, rRNA

## Abstract

Since the framing of the Central Dogma, it has been speculated that physically distinct ribosomes within cells may influence gene expression and cellular physiology. While heterogeneity in ribosome composition has been reported in bacteria, protozoans, fungi, zebrafish, mice and humans, its functional implications remain actively debated. Here, we review recent evidence demonstrating that expression of conserved variant ribosomal DNA (rDNA) alleles in bacteria, mice and humans renders their actively translating ribosome pool intrinsically heterogeneous at the level of ribosomal RNA (rRNA). In this context, we discuss reports that nutrient limitation-induced stress in *Escherichia coli* leads to changes in variant rRNA allele expression, programmatically altering transcription and cellular phenotype. We highlight that cells expressing ribosomes from distinct operons exhibit distinct drug sensitivities, which can be recapitulated *in vitro* and potentially rationalized by subtle perturbations in ribosome structure or in their dynamic properties. Finally, we discuss evidence that differential expression of variant rDNA alleles results in different populations of ribosome subtypes within mammalian tissues. These findings motivate further research into the impacts of rRNA heterogeneities on ribosomal function and predict that strategies targeting distinct ribosome subtypes may hold therapeutic potential.

This article is part of the discussion meeting issue ‘Ribosome diversity and its impact on protein synthesis, development and disease’.

## 1. Introduction

Ribosomes were discovered by electron microscopy in the mid-1950s by virtue of their enormous size in comparison to other cellular components [1]. Ribosomes were later revealed as the catalyst of messenger RNA (mRNA) translation following the development of systems for *in vitro* protein synthesis [2–4]. Mechanistic investigations later solidified that ribosomes synthesize protein by directionally transiting mRNA in discrete, three-nucleotide codon steps using specific aminoacylated transfer RNA (aa-tRNA) substrates [5]. This conserved protein synthesis mechanism defines the genetic code linking mRNA and protein sequence in all organisms [6–8].

Ribosomes are ubiquitous two-subunit, megadalton-scale RNA–protein complexes typically present at high concentrations to support the protein synthesis capacities necessary for cellular homeostasis and growth [9,10]. Bacterial ribosomes are comprised of 3 rRNAs (5S, 16S and 23S) and 55 distinct ribosomal proteins (RPs), and they are ca 2.3 MDa in mass; while cytoplasmic mammalian ribosomes are comprised of 4 rRNAs (5S, 5.8S, 18S and 28S) and 80 distinct RPs, and they are ca 4.3 MDa in mass [11]. The globular domains of RPs decorate the periphery of the assembled ribosome while flexible extensions reach toward the ribosome’s core, which is principally comprised of intricately folded rRNAs [12,13]. The functional centres of the ribosome responsible for mRNA decoding and peptide bond formation are highly conserved across species and reside on the small and large ribosomal subunits, respectively [14]. Advances in structural biology have revealed that both functional centres are composed principally of rRNA, in line with the hypothesis that ribosomes are RNA catalysts and represent the most complex ribozyme presently known [15–17]. These observations support a model in which the primordial self-replicating proto-ribosome co-evolved with exogenous translation machinery over billions of years to meet modern cellular protein synthesis demands [18–20].

Rapid and faithful protein synthesis is achieved through a highly orchestrated series of transient interactions between aa-tRNA and protein translation factors with the ribosome at each codon increment [21]. The ribosome’s capacity to define the genetic code hinges on its capacity to differentiate, within just a few milliseconds, the interactions of cognate aa-tRNA with the mRNA codon located within the aminoacyl (A) site on the ribosome’s leading edge from those that are near cognate [22–24]. Said differently, the mechanism of translation hinges on specificities originating from the recognition of single nucleotide polymorphisms (SNPs) in mRNA. The sensitivity of the protein synthesis mechanism to such small perturbations provides context for understanding how variations in ribosome composition, including changes in rRNA sequence, RP composition or in post-transcriptional and post-translational modifications (PTrMs and PTMs, respectively) could exert mechanistic impacts [23,25,26]. While alterations of these kinds may globally affect the rate, efficiency and fidelity of protein synthesis in the cell, the salient question we attempt to address is whether physical heterogeneities within the ribosome pool can programmatically modify gene expression in a manner that supports fitness advantage.

The prevailing view that ribosomes are uniform, housekeeping assemblies that are only passively involved in regulating gene expression is grounded by observations that (i) ribosomes within a given organism are globally similar in composition, (ii) they have the capacity to translate highly diverse pools of mRNA [,^27^–^29^] and (iii) the translation mechanism is globally conserved [30]. However, evidence of ribosome heterogeneity is pervasive across vast phylogenetic distances. While RPs are generally encoded from single loci in the genome and are stoichiometrically present within individual ribosomes, RP paralogues exist and ribosomes with varied RP compositions have been documented to influence protein translation [26,31–36]. By contrast, individual organisms typically possess multiple competent ribosomal DNA (rDNA) operons that have the potential to encode distinct rRNA genes [37–41]. Sequence variations within each operon’s rDNA genes render an organism’s ribosome pool inherently heterogeneous at the level of rRNA [41,42]. Here, we offer a perspective on the potential contribution, significance and impact of genomically encoded rRNA sequence variation. Excellent reviews on additional layers of ribosome heterogeneity arising from PTMs of RPs and PTrMs of rRNAs, as well as RP stoichiometry and sequence, can be found elsewhere [31,43–47]. Given that taxonomic classification of species can be robustly demarcated based on sequence variations present within the rRNA genes of different organisms [48,49], we posit that rRNA heterogeneity within a single organism offers a potentially new means of defining cell type, or state, based on the variant rRNA alleles expressed.

Ribosome heterogeneities likely manifest as subtle changes to the ribosome’s energy landscape that alter the distribution of ribosome conformational states and the association and dissociation rates of transiently interacting cellular components [50,51]. Such changes may lead to altered initiation, elongation and co-translational folding rates that impact the translation efficiencies of distinct mRNA transcripts, or the sites of synthesis, through direct or indirect mechanisms [52–56]. Delineation of these links is only beginning to emerge through the implementation of highly sensitive approaches, including mass spectrometry, next-generation/third-generation sequencing, cryogenic electron microscopy (cryo-EM) and single-molecule imaging methods [40–42,57,58]. Here, we provide overviews of the encoding and maintenance of rRNA genes within the genomes of prokaryotic and eukaryotic species to provide context for general readers. We then turn to rRNA-encoded heterogeneities evidenced in prokaryotic and mammalian ribosomes, highlighting a case study in *Escherichia coli* that demonstrates how differential expression of conserved rRNA variant alleles can programmatically alter gene expression and phenotype. In this context, we emphasize the finding that ribosomes derived from different rRNA genes can be differentially targeted by small-molecule drugs. Given these data, we speculate that cellular transformations accompanied by changes in rDNA operon expression offer the potential to target ribosome ‘sub-types’ within distinct cell lineages, including cancers, with small-molecule interventions for therapeutic benefit.

## 2. rDNA operon multiplicity

Cell size and division are tightly coupled to the production of fully assembled ribosomes, which represent approximately 50% of a cell’s dry mass [59–62]. In actively growing cells, nearly 60−80% of a cell’s total transcriptional capacity is devoted to ribosome biogenesis [63]. In rapidly growing yeast cells, assembled ribosomes are produced and exported from the nucleus at an estimated rate of 2000−4000 per minute [59,64]. Given estimated cell growth doubling times and the number of ribosomes in different cell types (*E. coli* (20 minutes); approx. 50 000); (yeast (60 minutes); approx. 250 000); (mammalian (24 hours); approx. 5 000 000) [65–68]), this rate of production is likely conserved across the domains of life. Maintaining a balance in the production rates of ribosome components (rRNAs and RPs) is critical in both prokaryotic and eukaryotic cells. This is demonstrated by the observation that imbalances in the synthesis rates of ribosomal components in mammals couple to p53 pathways that can give rise to cell cycle arrest, and, if not rectified appropriately, cell death [59,69–72].

**Figure 1.**
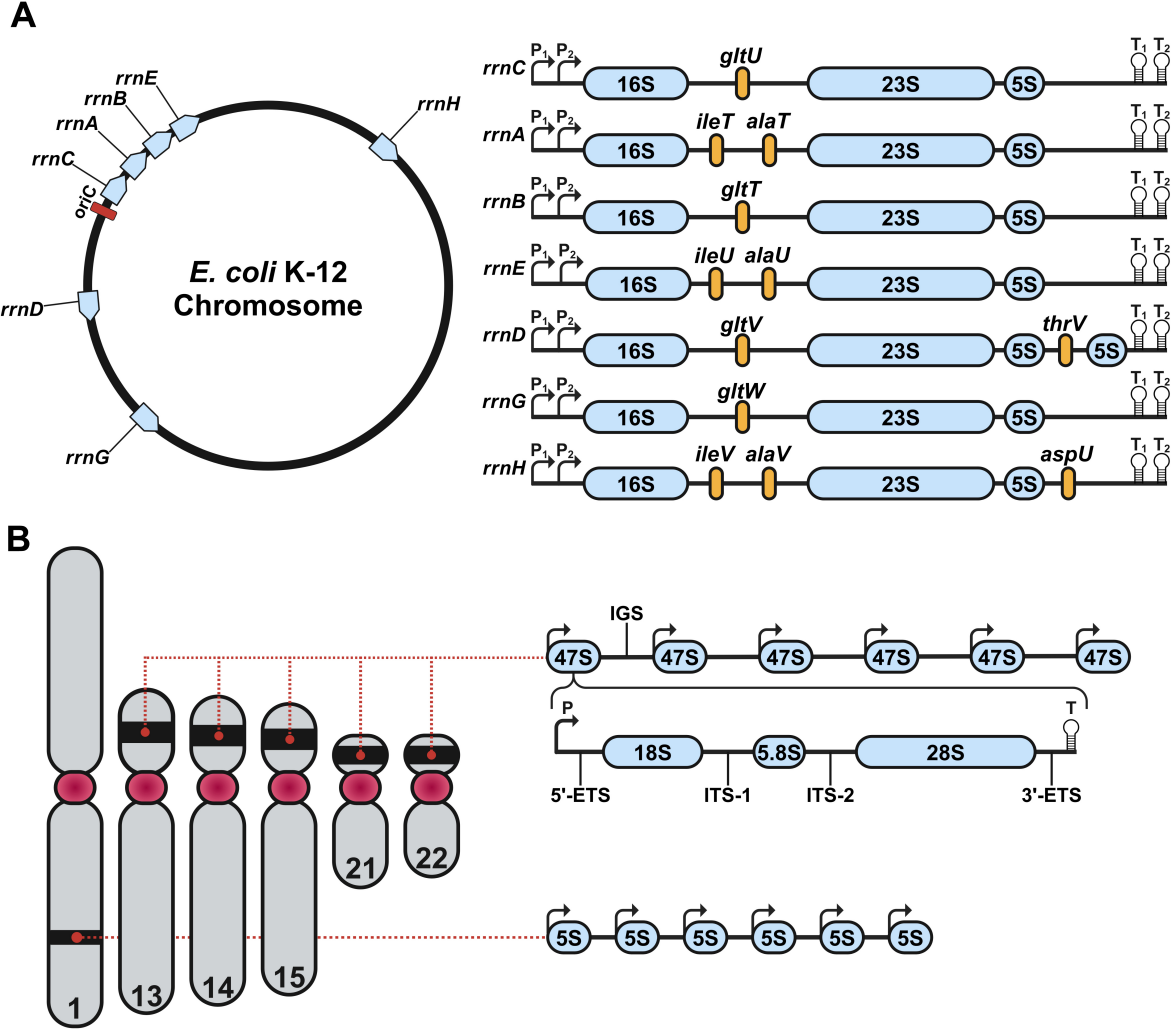
Genomic organization of rDNA in *E. coli* and human. (A) Chromosomal map of the seven rDNA operons *(rrn*C, *rrn*A, *rrn*B, *rrn*E, *rrn*D, *rrnG* and *rrnH*) in *E. coli*. On the left, each operon’s position is shown in blue relative to the origin of replication (oriC, red). On the right appear genetic structures of the seven rDNA operons. Each rDNA operon consists of the 16S, 23S and 5S rRNA genes. *rrnD* contains a second 5S gene, whereas all other operons only encode a single copy of each component. The rRNA genes are separated by internally transcribed regions, as well as unique tRNAs (yellow). Transcription initiation sites are marked by P₁ and P₂, termination sites for transcription are marked by T₁ and T₂. (B) Genomic organization of the rDNA operon in the human genome. Chromosome 1 encodes tandem repeats of 5S rRNA. Chromosomes 13, 14, 15, 21 and 22 encode tandem arrays of 47S rDNA operons within their p-arms. Each human rDNA operon contains a promoter (P), a 5’ externally transcribed region (5’-ETS), the 18S rRNA, internally transcribed region 1 (ITS-1), the 5.8S rRNA, internally transcribed region 2 (ITS-2), the 28S rRNA, the 3’ externally transcribed region (3’-ETS) and a transcriptional termination motif (T). Furthermore, each operon is separated by an intergenic spacing region (IGS). Created with BioRender.com.

Most organisms meet ribosome production and protein synthesis demands by encoding multiple competent rDNA loci. For example, typical laboratory strains of *E. coli*, such as MG1655, encode seven rDNA operons in their genomes (figure 1A) [73,74], while mice possess an average of *ca* 200 rDNA operons, encoded on chromosomes 12, 15, 16, 18 and 19 [41]. Human rDNA operons are encoded on the p-arms of the five acrocentric chromosomes 13, 14, 15, 21 and 22, with recent studies suggest that the average human genome encodes *ca* 300–500 rDNA operons (figure 1B) [75–77]. Hence, the cell’s burden of generating *ca* 3000 ribosomes per minute can potentially be distributed among all transcriptionally active rDNA operons, such that each operon produces just 6−10 ribosomes per minute. However, the rate of ribosome biogenesis may not be uniform across loci if their transcriptional promoters exhibit different strengths or regulatory control or if specific rDNA operons are epigenetically silenced. In actively growing *E. coli,* each of the seven rDNA operons is transcriptionally expressed at different levels and their relative and absolute expression levels can vary with growth conditions [42]. In differentiated mammalian cells, *ca* 50% of rDNA operons are transcriptionally silenced [78–81], but can become activated in the presence of stimulus or upon dedifferentiation [82–84]. As discussed below, the existence of conserved sequence variations in the structural rRNA genes in *E. coli*, yeast, mice and humans [76,85–87] specifies that differential expression of distinct rDNA alleles regulates rRNA heterogeneity in the actively translating ribosome pool.

## 3. Global organization of rDNA

The architecture of rDNA operons and the process of ribosome biogenesis are globally conserved across species. In all organisms, an RNA polymerase transcribes a single pre-rRNA molecule from each rDNA operon, which is then co-transcriptionally and post-transcriptionally processed into the distinct, fully modified rRNAs that associate with RPs [88]. In both prokaryotes and eukaryotes, rRNA processing involves hundreds of cellular factors that facilitate transcription, nucleolytic processing, folding and the incorporation of PTrMs*. E. coli* ribosomes contain *ca* 33 PTrMs, while human ribosomes contain ca 230 PTrMs [89–91]. Observed differences in PTrM patterns within the ribosome across tissue types and disease states suggest that PTrMs contribute to the physical heterogeneities present in translating ribosomes within the cell [92–94]. Excellent comprehensive reviews on ribosome biogenesis can be found elsewhere [59,88].

In bacteria, RNA polymerase, in association with the sigma 70 transcription factor (σ70), generates a single pre-rRNA transcript (30S) from each rDNA operon that contains the 16S, 23S and 5S rRNAs as well as one or more specific tRNAs (figure 1A, right panel) [87]. The pre-rRNA transcript is co-transcriptionally and post-transcriptionally processed through a multistep mechanism to generate the mature rRNA genes that cooperatively assemble with 50 RPs into the functional ribosomal subunits (30S and 50S) as well as mature tRNA [95]. The architecture of the human rDNA operon follows a globally comparable arrangement (figure 1B). Each operon is transcribed by the RNA polymerase I complex (Pol I), in concert with a host of associated transcription factors, to generate a 47S pre-rRNA transcript [96]. Co-transcriptional processing of the 47S pre-rRNA gives rise to three distinct rRNAs—18S, 5.8S and 28S rRNAs—which cooperatively assemble with 80 RPs that are regulated by the RNA polymerase II complex (Pol II) transcription, into the functional ribosomal subunits (40S and 60S) [97–99]. Unlike prokaryotes, tRNAs and 5S rRNA genes are not present within the 47S rDNA operons and are instead encoded elsewhere in the genome. The dominant cluster of 5S rRNA genes in the human genome is encoded on chromosome 1 (figure 1B) [100]. Both tRNAs and 5S rRNAs are transcribed by the RNA polymerase III complex (Pol III). Consequently, eukaryotic ribosome biogenesis requires regulatory crosstalk between Pol I, Pol II and Pol III transcription. Ribosome biogenesis in eukaryotes also differs from prokaryotes in that it occurs in phase-separated condensates called nucleoli within the nucleus. Furthermore, rather than installing PTrMs using specific proteins, 2’-O-methylations and pseudouridines are installed by C/D box and H/ACA small nucleolar RNA-protein complexes (snoRNPs or snoRPs), respectively, whose specificity is dictated by base pairing interactions with their target rRNAs [81,101–105].

## 4. rRNA heterogeneity in model organisms

Next-generation sequencing technologies that enable whole genome assemblies have revealed that a majority of organisms harbour multiple genetically distinct rDNA operons [106–108]. Variable regions in *E. coli* rRNAs reside at the solvent-accessible periphery of the assembled ribosome distal to the catalytic centres, an observation that has been largely interpreted as an indication of inconsequential genetic drift. Only recently has it been posited that variable regions within rRNAs potentially impart functional distinctions to assembled ribosomes [42,109].

The earliest evidence that conserved variable regions in rRNA may confer fitness advantages came from studies of rRNA expression patterns in extremophiles [108]. Sequence divergence in 16S, 23S and 5S genes within two rDNA operons from the halophilic archaea *Haloarcula marismortui* was reported to increase viability across different salt concentrations [110]. Analogous hypotheses have been put forward for *Thermobispora bispora,* wherein condition-specific expression of variant 16S rDNA alleles differentiated by 98 SNPs have been documented [111]. The malaria parasite *Plasmodium falciparum* also exhibits varied rRNA expression in its asexual and sporozoite stages, with substantial differences in the expansion segments and GTPase activating centres between the variant alleles that are of structural and functional importance [112–117]. Most recently, zebrafish have been found to express distinct rRNA subtypes during embryogenesis and in adult tissues [118]. While these studies demonstrate the existence of rRNA heterogeneity in distinct organisms, these observations raise many new questions. For instance, how might the expression of different alleles be regulated in response to different stimuli? What are the functional impacts of distinct rRNA alleles on physiology, and on what time scales do they affect the organism?

Efforts to probe the function of rDNA sequence variation in *E. coli* were initially attempted through genetic deletion of individual operons. These efforts ultimately led to the generation of an *E. coli* strain lacking all seven chromosomal rDNA operons (Δ7prrn) supported by one or more specific rDNA alleles expressed from a plasmid [119]. Experiments with Δ7prrn strains demonstrated that all seven rDNA operons could support life, although phenotypic differences in cell growth were observed, most notably for the *rrnH* operon [120,121]. This system proved crucial to recent investigations examining the longstanding question of how the seven unique operons are differentially regulated in response to environmental stimuli as well as the functional impacts of variable regions within the *E. coli* ribosome [42].

## 5. Nutrient limitation-induced changes in rDNA operon expression in *E. coli*

The first evidence that ribosome heterogeneity resulting from the expression of distinct rDNA alleles can exert programmatic physiological consequences was demonstrated by Kurylo *et al*. [42]. The authors of this study employed paired-end, short-read Illumina sequencing to show that all seven rDNA operons in the common laboratory *E. coli* strain MG1655 are natively expressed at approximately equal levels in a rich growth medium. Control experiments using defined mixtures of distinct rRNA alleles confirmed the accuracy of this approach. As expected for general stress response induction, they further showed reductions in global rDNA expression and translation upon nutrient limitation. Unexpectedly, however, the expression levels of each rDNA operon were reduced to different extents. Expression from operons close to the origin of replication were static (*rrnC*) or reduced (*rrnA*, *rrnB* and *rrnE*), while distal rDNA operons had increased expression levels (*rrnD*, *rrnG* and *rrnH*) on a relative basis. The expressed variant pool exhibited >98% correlation with the rRNA variants present in the actively translating polysome pool. These observations revealed that the actively translating ribosome pool in *E. coli* is intrinsically heterogeneous at the level of rRNA and can change in response to nutrient limitation-induced stress.

**Figure 2.**
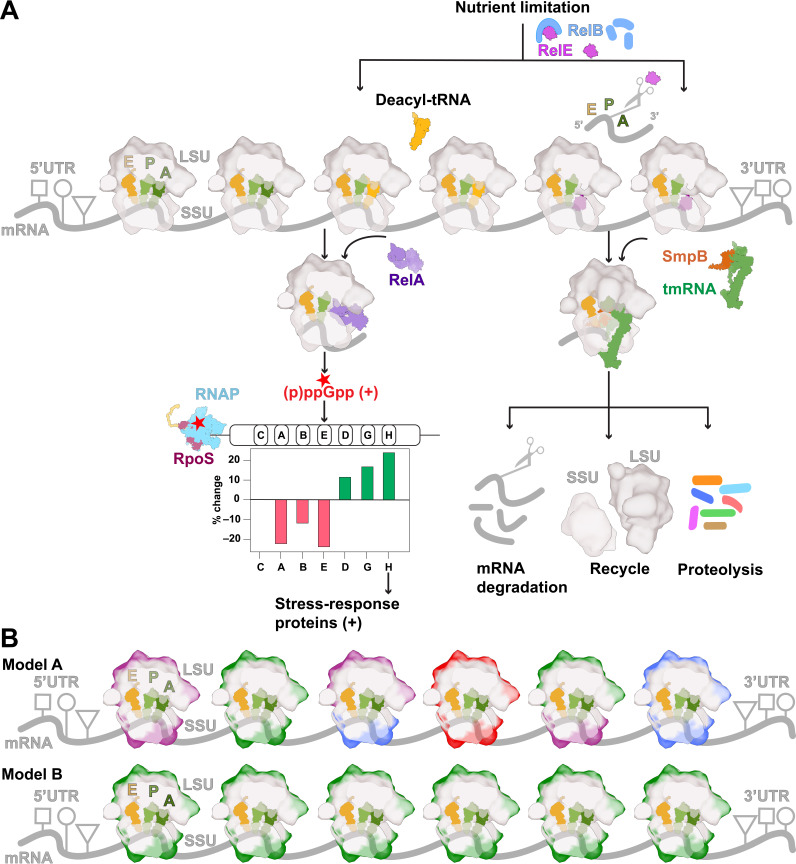
Model of ribosome heterogeneity in *E. coli*. (A) Two distinct pathways for releasing stalled ribosomes during nutrient-limited stress. The first pathway is the stringent stress response, where ribosomes stalled on mRNA bind deacylated tRNA at the A site, thereby facilitating the association of RelA (Protein Data Bank (PDB): 5KPV). This interaction induces the production of (p)ppGpp, an alarmone that ultimately results in elevated transcription of rrnH carried out by RNA polymerase (PDB: 5MY1) and RpoS (PDB: 6UUC) complex within the cell. Consequently, the population of stress-response proteins is upregulated as a direct consequence of nutrient-limiting conditions. The alternative pathway is induced upon degradation of RelB in the RelB–RelE complex, which releases RelE (PDB: 4V7J) to bind at the A site of stalled ribosomes in the absence of aa-tRNA. RelE cleaves mRNA at the second nucleotide of the A site codon, leading to the association of small protein B (SmpB) and the tmRNA complex (PDB: 7AC7), which ultimately releases stalled ribosomes from the mRNA. (RNAP, RNA polymerase) (B) In the cartoon diagram, different colour patches indicate that the ribosomes are composed of different variants of 16S and 23S rRNAs. Model A illustrates mRNA translation by a heterogeneous population of ribosomes, each carrying different rRNA derived from various rDNA operons. Model B illustrates mRNA translation by a homogeneous population of ribosomes originating from a single rDNA operon. The shapes shown in the mRNA cartoons depict the PTrMs present in 5’ and 3’ untranslated regions (UTRs). (Key: LSU: large subunit, SSU: small subunit)

Canonical nutrient limitation-induced stress response pathways are initiated by deacylated tRNA binding to the A site, which activates stress response proteins RelA and RelE and recruits them to the ribosome (figure 2A). Upon binding to the ribosome, RelA synthesizes the ‘magic spot’ alarmone (p)ppGpp to impart global transcriptional and translational changes in the cell that reduce expression of energy-intensive gene sets and initiate general stress response pathways [122] (figure 2A). Transcriptional changes are mediated in part by the direct binding of (p)ppGpp to RNA polymerase, which reduces its affinity for the promoters of housekeeping genes, including rRNA, tRNA and motility-related genes [123]. Once liberated, RNA polymerase is redirected to stress response gene promoters via its association with RpoS (σ38), a ‘master’ regulator of the general stress response [42,124] that is otherwise rapidly degraded by the ClpXP protease [125]. Nutrient limitation-induced stress also activates RelE via proteolytic cleavage of its inhibitor RelB. This allows RelE to bind and cleave mRNA within translating ribosomes, leading to the termination of protein synthesis and proteolytic degradation of truncated protein products via the transfer-messenger RNA (tmRNA) rescue pathway (figure 2A) [126].

Based on *in vitro* studies of RNA polymerase binding affinity for distinct rDNA promoters and the changes in rRNA expression observed upon nutrient limitation [42], it was hypothesized that RNA polymerase more readily liberates from the promoters of *rrnA*, *rrnB* and *rrnE* operons upon (p)ppGpp binding, thereby giving rise to a relative increase of *rrnD*, *rrnG* and *rrnH* ribosomes in both the total and actively translating ribosome pool (figure 2A). These findings, which were later validated by orthogonal sequencing methods [42,127], suggest that operons distal to the origin of replication may be evolutionarily conserved in both their genomic position relative to other operons and their sequence variation so as to maintain fitness against nutrient limitation-induced stress.

## 6. Allelic variation in bacterial 16S rRNA alters gene expression and phenotype

The *rrnH* operon—the most highly upregulated operon on a relative basis upon nutrient limitation-induced stress in *E. coli* strain MG1655—possesses 10 SNPs in the small subunit head domain of the *rrsH* (16S) gene, 9 of which cluster in the helix 33 element within variable region 6 (h33; V6) [42]. These h33 SNPs are evolutionarily conserved across the *Enterobacter* genus, members of which exhibit feast-to-famine lifestyles [42]. This finding argues that the h33 sequence variation within the *rrsH* gene confers fitness advantages related to nutrient limitation-induced stress. Support for a causal relationship was found through investigations showing that raising *rrsH* expression levels via exogenous expression from a plasmid upregulated RpoS protein levels and the nutrient limitation-induced stress response in a dose-dependent manner [42].

Motivated by these observations, a comparative study was performed on Δ7prrn strains transformed with plasmids expressing either the full *rrnB* operon (BBB) or an altered *rrnB* operon bearing the 10 small subunit head domain SNPs present in the *rrsH* (16S) gene of the *rrnH* operon (HBB). Δ7prrn strains transformed with BBB and HBB plasmids grew at similar rates in both rich and minimal media. However, a comparative analysis of the transcription profiles of these strains grown in minimal media revealed that *ca* 20% of the transcriptome was changed by the *rrsH* variant allele [42]. Notably, the altered gene set, including consideration of the direction of change, showed significant overlap with genes programmatically linked to the general stress response [126]. These included the regulons for the (p)ppGpp alarmone-producing RelA protein as well as RpoS. Hence, HBB ribosome expression facilitates upregulation of canonical pathways of the nutrient limitation-induced stress response [124–126]. How this occurs mechanistically remains an open area of investigation.

Notably, genetic interaction studies showed that RelA and RelE, as well as the metabolic enzyme and virulence-associated protein alcohol dehydrogenase (AdhE), functionally interact with the *rrsH* variant SNPs [42]. This finding led to the conclusion that HBB ribosomes are likely to interact differentially with regulatory factors, including translation factors that govern protein synthesis, to influence gene expression [42]. As the expression of HBB ribosomes did not change RpoS mRNA levels, while RpoS protein levels increased [42], the relationship between HBB expression and the stress response was posited to include post-transcriptional regulation mechanisms, which are well documented for RpoS [125]. In this context, two globally distinct models of post-transcriptional regulation should be considered. In one model (Model A), ribosomes composed of different rRNA load onto all mRNAs equivalently to promote global changes in translation fidelity, which gives rise to an accumulation of misfolded or unfolded proteins that compromise the proteolytic degradation of RpoS (figure 2B) [128,129]. In a second model (Model B), HBB ribosomes preferentially initiate on, or elongate, RpoS mRNAs (figure 2B). Model B is grounded by knowledge that the upregulated gene set associated with the HBB ribosome expression is statistically enriched for rare codons [42] and that RpoS mRNA contains 11 rare codons (the most in *E. coli*) that positively regulate RpoS protein levels [130]. In this context, it was noted that if *rrsH* variant ribosomes read through rare codons more efficiently, this would reduce mRNA susceptibility to RelE cleavage and prevent tmRNA-mediated rescue of ribosomes translating RpoS mRNA to increase RpoS expression [130]. While further experiments are needed to address these and other models, the validity of Model B could be assessed through experiments designed to query the distribution of ribosome sub-types bound to RpoS mRNAs in the actively translating ribosome pool (figure 2B).

## 7. Variant ribosomes exhibit altered drug sensitivity and structure

**Figure 3.**
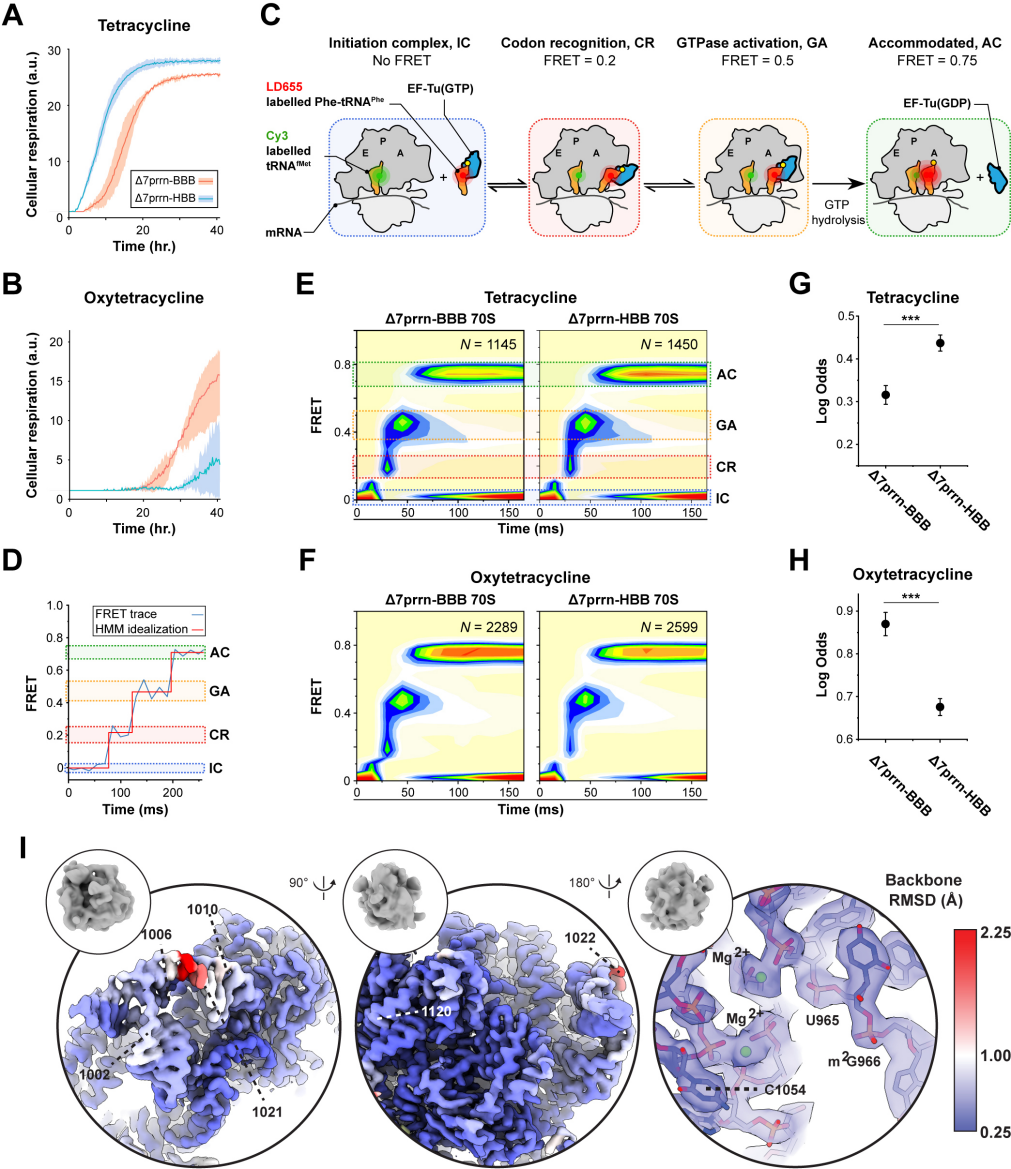
Variant ribosomes exhibit altered drug sensitivity. (A,B) BIOLOG phenotypic screening comparing the growth of Δ7prrn-BBB (red) and Δ7prrn-HBB (cyan) in the presence of (A) tetracycline and (B) oxytetracycline. Panels A and B are modified from Kurylo *et al. [*42]. (C) Schematic of tRNA selection measured using smFRET. FRET is monitored upon delivery of a ternary complex consisting of LD655-labelled Phe-tRNA^Phe^ in complex with GTP bound elongation factor thermo-unstable (EF-Tu(GTP)) to surface-immobilized initiation complexes containing Cy3-labelled tRNA^fMet^ in the P site. Incoming aa-tRNA enters the A site through a series of distinct intermediates; codon recognition (CR, FRET = 0.2), GTPase activation (GA, FRET = 0.5) and finally reaches the fully accommodated state (AC, FRET = 0.75). (D) Representative smFRET trace illustrating accommodation of incoming aa-tRNA into the A site through the intermediate steps of tRNA selection. (E,F) Post-synchronized population histograms of smFRET tRNA selection events on BBB and HBB ribosomes isolated from Δ7prrn-BBB and Δ7prrn-HBB respectively, in the presence of (E) tetracycline and (F) oxytetracycline. (G,H) Log odds of incoming Phe-tRNA^Phe^ reaching the accommodated state in the presence of (G) tetracycline and (H) oxytetracycline. (I) PDB 7N1P was fitted into the head focused-refined cryo-EM maps of the BBB and HBB ribosomes, and the two maps were aligned based on the core of the head domain. The backbone root mean squared deviation (RMSD) between BBB and HBB ribosome structures was calculated in Chimera and the BBB ribosome cryo-EM unsharpened map is coloured according to their backbone deviations. Left and middle panels show two views encompassing the sequence variants, while the right hand panel shows the tetracycline binding site.

To gain a deeper understanding of how variant rRNA alleles facilitate the nutrient limitation-induced stress response, Kurylo *et al*. employed BIOLOG phenotypic microarrays to compare the relative fitness of cells expressing BBB or HBB ribosomes under a range of growth conditions [42]. These assays revealed that cells expressing *rrsB-* or *rrsH*-bearing ribosomes (∆7prrn-BBB and ∆7prrn-HBB, respectively) exhibit disparate resistance profiles *in vivo* to various tetracycline-class antibiotics (figure 3A,B). Tetracyclines bind directly to the small subunit head domain of the ribosome at a region distal to h33 [131] and are widely employed in clinical settings to combat infectious disease [132].

To assess whether the observed differences in tetracycline-class antibiotic sensitivity were directly related to physical alterations in the ribosome, isolated BBB and HBB ribosomes were examined for protein composition and rRNA structure using comparative proteomics and dimethyl sulfate (DMS) mutational profiling with sequencing (DMS-MaPseq,) respectively. By these methods, BBB and HBB ribosomes were shown to be indistinguishable in terms of RP composition and global rRNA structure. However, ribosomes isolated from ∆7prrn-HBB were found to preferentially associate with AdhE, consistent with a direct interaction between AdhE and the rrsH variant SNPs and the notion that variation in rRNA sequence can result in altered interactions with cellular factors.

To measure the comparative impact of tetracyclines directly, mRNA decoding by BBB and HBB ribosomes was measured *in vitro* using single-molecule fluorescence resonance energy transfer (smFRET) [133,134]. Here, the rate and efficiency of tRNA selection by BBB and HBB ribosomes were quantified via the changes in FRET associated with the delivery of acceptor-labelled (LD655) Phe-tRNA^Phe^ by thermally unstable elongation factor (EF-Tu) to ribosomes bearing donor-labelled (Cy3) tRNA^fMet^ (figure 3C). This multi-step process is typified by transient low-FRET binding events (codon recognition, CR, FRET = 0.2) where aa-tRNA samples the mRNA codon presented in the ribosomal A site, followed by forward progression to an intermediate state where EF-Tu is primed for GTP hydrolysis (GTPase activation, GA; FRET = 0.5) and finally to a stable high FRET fully accommodated state (accommodated, AC; FRET = 0.75), where peptide bond formation occurs (figure 3C,D). Consistent with the *in vivo* susceptibility profiles observed [42], these *in vitro* studies showed that BBB ribosomes are comparatively sensitive to tetracycline-mediated inhibition of tRNA selection, while HBB ribosomes are comparatively sensitive to oxytetracycline (figure 3E–H) [135]. Hence, endogenously encoded SNPs within h33 (V6) of the small subunit of the ribosome alter either ribosome structure or dynamics in a manner that allosterically impacts the actions of tetracycline-class antibiotics on tRNA selection at a distance. Supporting this conclusion, mutations within the SSU head domain distal to the tetracycline binding site can confer either resistance or hypersensitivity to tetracycline [136].

To gain further insight, cryo-EM studies were performed on isolated BBB and HBB ribosomes (figure 3I). These data revealed altered flexibilities of h33 in BBB and HBB ribosomes such that comparison of their atomic models showed that the *rrsH* variants give rise to local structural changes in the regions of sequence variation (figure 3I, left and middle panels). However, quantifiable structural differences in tetracycline binding sites could not be ascertained (figure 3I, right panel). This finding suggests that sequence variation within h33 exerts relatively subtle structural or dynamic impacts on the small subunit that alter how BBB and HBB ribosomes interact with tetracycline-class antibiotics. Nonetheless, the observed differences in tetracycline antibiotic sensitivities for these ribosome sub-types hold potentially profound implications. In addition to informing strategies to more specifically target pathogenic *E. coli* in which all rDNA operons encode *rrnH* variant alleles [137,138], this knowledge also provides a potentially generalizable proof-of-concept that ribosome heterogeneities may be therapeutically targeted.

## 8. rRNA heterogeneity in eukaryotes

Variations in human rDNA operons, both in their genomic organization and specific sequences, have been observed in multiple eukaryotic species [41,102,139–141]. Intra-individual rDNA copy number can also vary between members of the same species [41,85] and across generations [142]. Conserved sequence variation has been observed in insects [143], mammals [39,144] and plants [145], particularly in intergenic regions that interdigitate between the three rDNA genes of the pre-rRNA [146]. However, rigorous characterization of the natural variance in rDNA across the human population had been difficult to achieve with historically small sets of sequenced human genomes.

**Figure 4.**
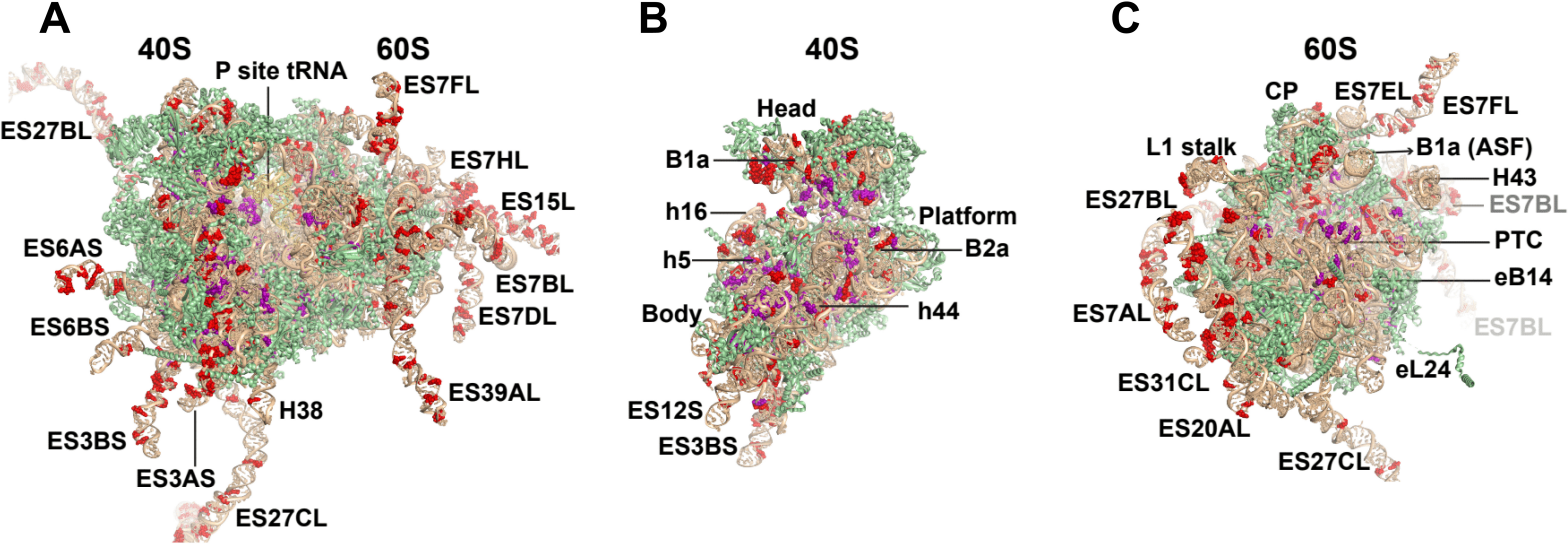
High-frequency rRNA variants detected in humans. (A) 80S human ribosome with modeled expansion segments labelled at high-frequency allelic variant positions (>20%). (B) 40S small subunit of human ribosome with high-frequency variant alleles. (C) 60S large subunit of human ribosome with variant high-frequency variant alleles. Key structural features of the ribosome are indicated. Key: rRNA, tan; RP, green; variant nucleotide, red; posttranscriptional modifications, purple. Model based on PDB: 8G5Y. Gray labels of expansion segments indicate their relative distance from the viewers perspective.

Parks *et al.* [41] overcame this shortcoming by performing a population genetics investigation of the 2504 genomes established through the 1000 genomes project (1KGP) to assess whether humans possess varied rDNA operon copy numbers and rRNA sequence variation [41]. In this investigation, paired-end Illumina reads were pulled from publicly available PCR amplified DNA libraries by computational hybridization to a prototype rDNA operon sequence. Eight terminal bases at either end of the reads were trimmed to eliminate potential low-quality bases, thereby improving overall mapping quality. Reads from this pool that mapped more precisely to the annotated human genome reference with BWA [147] and GATK [148] were also subsequently removed. Finally, unmapped reads from the previous step were mapped to a human rDNA ‘prototype’ to assess sequence variation within human rDNA operons using the LoFreq variant calling tool [149]. Correcting for read-depth deficiencies associated with GC-rich ribosome regions (approx. 60–80% GC across eukaryotic rDNA operons) [150], rDNA copy number was found to vary between individual humans by up to two orders of magnitude (61 to 1590 rDNA copies) [41]. Notably, stratifying rDNA copy number variations were present between superpopulations sharing common ancestral origins, suggesting evolutionary conservation of copy number levels within specific populations [41]. A total of 1790 variant alleles were identified occurring across 1662 distinct positions within the 7184 nucleotides that make up the 5S, 5.8S, 18S and 28S rRNA genes, the vast majority of which (1739) were identified as SNPs. A total of 497 variant alleles occurred within at least one individual at allele frequencies >20%, with the average individual encoding 32 high-frequency (>20%) variant alleles (figure 4; [41]). While short insertions and deletions (indels) were also identified, these data likely represent a lower limit on indel frequency given the known challenges associated with identifying such errors with relatively short read sequencing [151]. Accounting for read depth limitations arising from GC content, variant positions were overrepresented in 18S rRNA and underrepresented in 28S rRNA both per individual and across populations. SNP and copy number variations outside of rDNA regions (i.e. protein coding regions) stratify by ancestry [152,153]. In addition to stratifications based on rDNA copy number, the intra-individual allele frequency for 327 variants, spanning all four rRNA genes, also stratified by population [41]. Notably, 24 of the most highly population-stratified variants that also exhibited high-allele frequency clustered within regions of the ribosome previously associated with human disease, including Diamond-Blackfan anemia, body plan formation during development and cancer [154–157]. These findings suggest that human evolution is under pressure at the population level to preserve both rDNA copy number and sequence variation.

Interestingly, a total of 61 identified variants localized to 59 of the 256 sites in rRNA reported to exhibit PTrMs [43,158,159]. PTrM variability in rRNAs is prevalent in eukaryotes and can occur within single cells and across different cell types [89,160–162]. PTrMs have also been observed to vary throughout chondrogenic differentiation [163], lifecycle stages [164], temperature changes [165], brain development [166] and embryonic stem cell differentiation [166]. As PTrMs typically cluster within the core of the ribosome and contribute to both mRNA decoding and peptide bond formation, conserved sequence variation at PTrM sites may also functionally impact the process of translation [167]. Conserved rRNA sequence variations at sites of PTrMs further suggest that the expression of variant alleles may alter, or even disrupt, interactions, with the snoRNPs responsible for depositing PTrMs to impart physical distinctions in the ribosome biogenesis process.

To further assess the potential relevance of these findings, Parks *et al.* [41] analysed the rDNA copy number and sequence variation of 32 mouse strains targeted by the Mouse Genomes Project [168]. Although limited in comparison to the human 1KGP in terms of both the number of individuals and depth of coverage, this analysis detected 285 distinct variant alleles at 276 positions in mouse rRNA genes. Strikingly, 80 of these positions of variation (approx. 29%) were also present in humans, more than half of which exhibited the same reference and variant allele [41]. Many of these conserved variant alleles mapped to functional centres of the ribosome, including interaction sites of translation initiation [169] and elongation [170] factors.

**Figure 5.**
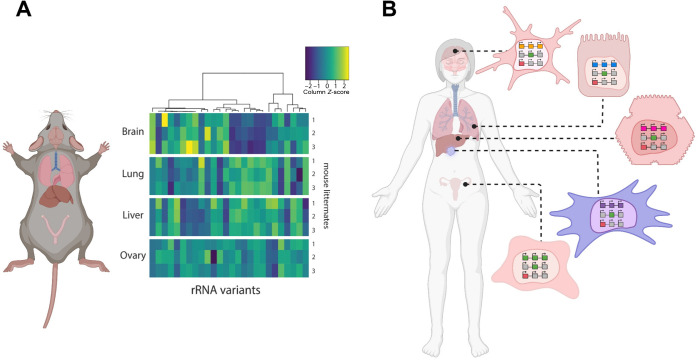
Tissue-specific expression of rRNA variants. (A) Experimentally observed variance in rRNA across different mouse tissues. This panel is reprinted from a previous publication [41] with the same caption: 'rRNA variant expression heat map and hierarchical clustering of the 26 variants detected to be differentially expressed among pairs of tissues. Each row represents a biological replicate. Rows are grouped by tissue source (three biological replicates, that is, rows, per tissue source). Each column represents an rRNA variant. Expression is normalized per rRNA variant (that is, by column) across all replicates and tissues (that is, 12 samples per each column). For example, the rRNA variant represented by the leftmost column has higher relative expression in brain, whereas the variant represented by the rightmost column has the lowest relative expression in liver.' (B) Model of tissue-specific rRNA variant expression in humans. Consistent with observations in mice, it is expected that humans also exhibit tissue-specific, and possibly even cancer-specific expression of rRNA variants from distinct loci (coloured squares in the nuclei of various tissues). Created with BioRender.com.

Parks *et al.* further performed and analysed in triplicate, paired-end RNA-seq data from brain, lung, liver and ovary from three mouse littermates to explore whether variant rDNA allele expression is present and conserved in these tissues [41]. RNA-seq reads were aligned against the rDNA prototype, variants were identified using LoFreq [171] and differential expression of variant alleles between pairs of tissues could be calculated. Despite the heterogeneity intrinsic to each tissue sample, this analysis identified 70 distinct positions of expressed mouse rRNA sequence variation that were conserved, 31 of which were also detected by paired-end rDNA sequencing. For these variants, intra-individual genomic and rRNA expression allele frequency were highly correlated. Of the 70 expressed rRNA variants, 26 exhibited tissue-specific rRNA expression (figure 5A) [41]. Each pair of tissues was distinguished by a subset of these differentially expressed variants. Fifteen differentially expressed variants were detected by paired-end Illumina sequencing of rDNA; four coincided with known positions of rRNA modification, including residue 1248 in the 18S rRNA that has been implicated in human cancer [172]; Five of the most differentially expressed variants mapped to expansion segment 27 of the 28S rRNA, which associates with protein folding machinery near the nascent peptide exit tunnel on the solvent side of the large ribosomal subunit [173–175]. In addition to being expressed in mouse tissues, polysomes isolated from the mouse epithelial cell line NMuMG demonstrated the presence of rRNA variants in the actively translating ribosome pool [41], in line with previous literature [39,176].

While the study by Parks *et al.* [41] was the first to report on the degree of variation in the human population at the level of rRNA, a series of additional investigations have been supported the notion that rDNA operons in mammals exhibit sequence variation. However, quantitative information on the specific variant frequencies differed. For instance, a recent study by Rothschild *et al.* [177], which was based on long-read Pacific Biosciences sequencing technologies, reported that rRNA variant alleles are predominantly (approx. 95%) represented by short indels. This is in stark contrast to Parks *et al.* [41], who reported that 97% of variants were SNPs. One reason for this discrepancy may relate to PCR and mapping algorithm challenges associated with short-read sequencing technologies, particularly in highly repetitive genomic regions. It may also be possible that there are nuances to Pacific Biosciences long-read sequencing technologies and/or the new variant calling pipeline, reference gap alignment (RGA), employed by Rothschild *et al.* [177], that have yet to be revealed. Discrepancies may also exist owing to differences in the computation workflows employed by the two groups. Pacific Biosciences long-read sequencing attempts to resolve inherent propensities for inappropriately calling indels by performing circular consensus sequencing (CCS) of each DNA fragment, akin to calling a consensus read from many distinct reads. With this approach, random noise can be systematically eliminated with increasing CCS read depth. However, if CCS read depth is insufficient, the output of RGA, which uses every read to produce a global, gap-aware reference, may inadvertenly overestimate indels. While disparities in short-read and long-read sequencing technologies have been previously noted [178], Rothschild *et al.* have used orthogonal means to validate sequence variations called with their pipeline [177]. As the precision of both methods is undoubtedly imperfect, future studies aimed at clarifying the true nature and extent of human rRNA genetic diversity will be of substantial interest to the field.

## 9. rRNA heterogeneity in human disease

Disorders known as ribosomopathies underscore the importance of proper ribosomal function for human health [179–184]. Ribosomopathies also emphasize the close relationship between RP haploinsufficiencies, ribosome biogenesis defects and carcinogenesis [183,185]. The association between ribosome biogenesis abnormalities and human disease was established concurrent with the advent of tools to visualize the number and size of nucleoli in the cell [186–190]. Increased nucleolar area correlates with increased rRNA expression in prostate [191], cervical [192] and colorectal [193] cancers. In addition to associations with cancer progression [194], changes in ribosome biogenesis have also been linked to cellular differentiation [195–197] and metastasis fuelled by the epithelial–mesenchymal transition [198]. Today, altered PTrM profiles arising from altered ribosome biogenesis are used to discriminate between cancer subtypes, tumour grade and mutational status [172,199–206].

The molecular bases of most ribosomopathies have been attributed with perturbations in RPs that affect the cell’s capacity to make ribosomes in sufficient quantities for cellular homeostasis [72,183]. Direct links between rRNA sequence variation and diseases remain to be established. However, evidence connecting rRNAs' PTrM status with different phenotypic and disease states is emerging [93,94,207]. Recent results also suggest that rRNA sequence and PTrM variations may contribute to carcinogenesis [177,207].

## 10. Outlook

The research findings presented here lead us to hypothesize that humans likely harbour tissue-specific rRNA variants and linked PTrM patterns that contribute meaningfully to human physiology (figure 5B). It follows that changes in the expression of distinct rRNA alleles—accompanied by potentially distinct ribosome biogenesis programs (e.g. altered snoRNP utilization)—could be associated with human disease states.

Programmatic changes in rRNA variant expression could be achieved through well-established epigenetic and genetic transcriptional control mechanisms to precisely regulate the differential expression of specific rRNA alleles in response to environmental stimuli. The regulation of post-transcriptional modifications may similarly arise via alterations in rRNA sequence variation coupled with distinctions in ribosome biogenesis. Both rRNA sequence variation and altered PTrM patterns could analogously impact RP association energies as well as the potentially vast number of protein and RNA components of the cell that transiently interact with assembled ribosomes [31]. In the absence of robust degradation programs to specifically degrade the ribosome pool in a ribosome subtype-specific manner, transcriptional changes in the distribution of rRNA variants in the ribosome pool would likely persist for multiple cell generations given ribosome half-life estimates of up to multiple weeks [208–210]. This phenomenon would be akin to the established transgenerational inheritance of phenotypes associated with small noncoding RNAs, wherein extranuclear RNAs facilitate the inheritance of epitranscriptomic information [211]. In this instance, the inheritance of ribosome sub-types could confer a fitness advantage by bestowing offspring with the translational machinery needed for a particular environment or stress.

We note in this context that ribosome heterogeneity may also play a role in determining cell-type specific impacts of translation- and ribosome-targeting drugs [212–214]. This consideration, combined with the potential specificity of antibiotics for distinct ribosome sub-types (e.g. tetracycline for HBB and oxytetracycline for BBB ribosomes; figure 3), provide a principled foundation for exploring whether small molecules may be employed in therapeutic settings to target specific disease states (figure 5B). While speculative, targeting variant ribosomes and/or the cellular components, including translation factors, that interact with them would represent a strategy analogous to effective treatments for infectious disease with small molecule drugs, in which ribosomes within the pathogen are specifically targeted while those of the host are unaffected.

Quantifying rRNA sequence and PTrM variations in human health and disease is therefore of potentially significant importance. Robust quantifications will undoubtedly require continued efforts to establish appropriate sequencing (i.e., Illumina, Oxford Nanopore or Pacific Biosciences Hi-Fi) and bioinformatic (i.e., BWA [147]; minimap2; LoFreq [149]; GATK [148]) workflows to address the challenges associated with highly GC-rich rDNA and rRNA elements [40,42,177]. Given that rDNA operons are known hotspots of damage and recombination [215,216], these sequencing efforts should consider focusing on tissues or primary cell lines rather than immortalized cell lines that have been highly propagated under selection pressures for rapid growth. To fully elucidate ribosomal heterogeneity, a multi-disciplinary approach integrating structural biology, functional genomics, biochemistry and advanced computational methods will likely be necessary. Concerted efforts on these fronts will help decode the complexities of ribosomal function in normal physiology and pave the way for targeted interventions in diseases where ribosomal dysregulation plays a critical role.

## Data Availability

Cryo-EM maps and atomic models were deposited at the Electron Microscopy Data Bank (EMDB) and RCSB Protein Data Bank (PDB), respectively, for the two bacterial ribosome structures: BBB-70S: EMD-48830 and PDB: 9N2B; HBB-70S: EMD-48831 and PDB: 9N2C. Supplementary material is available online [217].
